# Translation and Cross-Cultural Adaptation of the Cancer Health Literacy Test for Portuguese Cancer Patients: A Pre-Test

**DOI:** 10.3390/ijerph19106237

**Published:** 2022-05-20

**Authors:** Ana Barros, Helena Santos, Luís Moreira, Filipe Santos-Silva

**Affiliations:** 1 i3S—Institute for Research and Innovation in Health, University of Porto, Rua Alfredo Allen 208, 4200-135 Porto, Portugal; fsilva@ipatimup.pt; 2 IPATIMUP—Institute of Pathology and Molecular Immunology, University of Porto, Rua Júlio Amaral de Carvalho 45, 4200-135 Porto, Portugal; 3 FEP—Faculty of Economics, University of Porto, Rua Dr. Roberto Frias, 4200-464 Porto, Portugal; hsantos@fep.up.pt; 4 CITCEM—Transdisciplinary Research Centre “Culture, Space and Memory”, University of Porto, Via Panorâmica s/n, 4150-564 Porto, Portugal; 5 RECI—Research Unit in Education and Community Intervention, Piaget Institute, Av. João Paulo II, Lote 544, 2.º, 1950-157 Lisboa, Portugal; luis.moreira@ipiaget.pt; 6 Health School of Vila Nova de Gaia, Piaget Institute, Alameda Jean Piaget 106, 4405-678 Vila Nova de Gaia, Portugal; 7 FMUP—Faculty of Medicine, University of Porto, Alameda Prof. Hernâni Monteiro, 4200-319 Porto, Portugal

**Keywords:** cancer health literacy test, translation, cultural adaptation, cancer literacy

## Abstract

Assuming the multidimensionality of health literacy, new complex and comprehensive approaches are more adequate to specific disease contexts, such as cancer. Assessing cancer literacy levels is a priority, since it entails potential serious implications for disease outcomes and patient’s quality of life. This article reports on the translation and cultural adaptation of the Cancer Health Literacy Test to measure cancer literacy in Portuguese cancer patients. A multidisciplinary team of experts ensured the translation and cultural adaptation of the CHLT-30. A pre-test was conducted in two stages to evaluate the Portuguese version (CHLT-30 PT) in a sample of cancer patients (n = 71). Descriptive statistics were used to characterize the sample. Reliability (test–retest and internal consistency) and construct validity of CHLT-30 PT were assessed. The results obtained show a good internal consistency of the tool, respectively (Cronbach’s alpha = 0.86 in the test and 0.80 in the retest). Patients’ raw score mean in both test (23.96) and retest (25.97) and the distribution of scores categories are not statistically different. A suggestive association between higher education level and better total score was found compared to the results reported in CHLT-30-DKspa. The results obtained in the pre-test are favorable, and the instrument is now suitable for the next steps of the validation process. A Portuguese version of this tool will allow outlining patients’ cancer literacy along the cancer care continuum, enabling the identification and implementation of adequate socio-educational strategies with highly positive impacts on health outcomes.

## 1. Introduction

Literacy skills are determinant in a changing globalized world, with international organizations recognizing the importance of functional literacy in everyday life, as emphasized in the 2030 Agenda for Sustainable Development [1,2,3,4,5]. 

People’s knowledge, perceptions, behaviors, cultural context and socioeconomic status have been progressively accepted as relevant health determinants. This progress strengthened the relation between citizens and healthcare systems and brought to the stage the concept of health literacy [6]. According to the World Health Organization, the cognitive and social skills, which determine the motivation and ability of individuals to gain access, use and understand the information to promote good health [7], are the main skills of health literacy. Health literacy is a multidimensional concept evolving through different perspectives and distinct intervention models. 

The monopoly of available information is no longer restricted to traditional institutions, such as schools or families, and socialization processes have become highly diffuse through multiple arenas [8] where digital technologies play an important part (e.g., social networks) [9].

Consequently, health information today is practically unlimited but not necessarily understood, selected and mobilized to generate knowledge. 

In parallel, current models of healthcare are changing toward the active involvement of patients and families with professionals, aiming to develop patient-centered approaches that are expected to promote shared decision making, emphasizing the importance of adequate health literacy skills.

To characterize health literacy, health organizations need to combine extensive assessment tools [10,11] with a more comprehensive, in-depth and cross-cultural perspective, trying to integrate not only the patient sociocultural and economic context but also the interactions with the health professionals and the specificities of the healthcare system [12]. 

### 1.1. Health Literacy Multidimensionality and Specific Disease Contexts

It is widely accepted that health literacy is determined by socioeconomic factors (e.g., age, gender, education, income, social class and even cultural and religious beliefs). In this sense, health literacy embraces different skills (writing, reading, listening, speaking) and dimensions [13], three of the latter being commonly referred to as functional (oral and writing comprehension and numeracy skills), interactive (seeking health information) and critical (the use of health information to promote health and wellbeing) [14,15].

Assuming the multidimensionality of health literacy, new, more complex and comprehensive holistic approaches are more adequate for specific disease contexts—as in the case of cancer. 

Cancer must be a priority in health literacy research, given the present emotional, physical and financial impacts and the expected increase in incidence over the next decades due to population growth and aging, along with socioeconomic and lifestyle changes. In Portugal, data reveal that cancer constitutes the second cause of death [16] and only in 2020, the data available on Globocan (WHO) estimate that 0.6% of the Portuguese population will develop cancer [16,17]. An improvement of cancer literacy will be the best strategy to counteract the increasing cancer burden [18,19].

Health literacy assumes a crucial role for cancer patients, especially in terms of disease management, in order to increase the success of treatment and survivorship. The impact of any cancer disease is “for life”, i.e., patients will have to cope with a radical change in their lives. In addition, the burden of the disease implies, among others, complex psychological issues regarding widespread myths and beliefs, trust/distrust in health professionals and treatments, emotional and affective aspects. Cancer literacy has major importance not only for patients who need to make (sometimes long-term) decisions based upon the information given in the healthcare services, while at the same time being exposed to misinformation disseminated on social media and digital platforms, but also for society as a whole and individuals that might effectively act toward health promotion and disease prevention with the necessary skills, confidence and autonomy.

Assessing cancer literacy levels is thus a priority, since it has serious implications for risk behaviors, adhesion to cancer screening programs, stage diagnosis and quality of life [17,20] and the implementation of educational programs (e.g., school health educational programs) that promote behavioral changes [21].

### 1.2. Cancer Literacy: The Concept and Its Measurement

As noted above, health literacy incorporates an operationalization of its definition related to functional literacy and basic skills, such as the ability to read and write. The same occurs for cancer literacy when we try to describe the concept. The first attempt to elaborate an operational definition of cancer literacy refers to it as all the knowledge a layperson needs to possess to understand the information and advice the health system has to offer with regard to preventing, diagnosing and treating cancer. Based on this definition, it may be noted that the ability or the skills that we need to understand the information are transversal to all subjects in health, while knowledge is specific to a subject or context, in this case, of a particular disease, cancer. On the other hand, if we look at the multiple health literacy definitions, they are constructed in a broader sense, so a specialization of the concept according to a certain area might be more useful to understand the impact of literacy in health outcomes [15].

Low cancer literacy could have serious implications for an individual’s quality of life, especially concerning cancer risk behaviors, participation in cancer screenings and stage diagnosis. A cancer-literate person should be more able to seek preventive medical checkups and be susceptible to well-founded advice on disease prevention, which is a crucial issue for the health economy, improving public health resources. It is also a key aspect for disease management. Patients with a high level of cancer literacy will interact better with health professionals and increase treatment success [15].

In order to improve cancer literacy, it is necessary to identify all its determinants, such as knowledge, skills, information needs, so the first step is to understand what people really know about cancer and what are the cultural and social characteristics associated with literacy in cancer [15]. If we constrain the scope of health literacy and focus on a specific disease context, such as cancer, the dimensions that are currently assessed with the most common health literacy tools are quite different. In the cancer spectrum, and since recent therapeutic advances are evolving cancer into a status of chronic disease, there is an increasing concern regarding prevention, and specifically screening and behavior. There is strong evidence that more research is needed in order to improve the distinction between different contexts of disease and illness that could be useful to describe and manage some health-related behaviors, reinforcing the assumption that health literacy as a one-fit-all concept is too general and in part meaningless, so the focus must be on disease-context literacy and how we can measure it. 

In this scenario, there are several instruments, internationally available, to measure cancer literacy, which we will briefly present (see the Supplementary Materials, Table S1).

Cancer awareness measure (CAM), developed in the UK in 2007 and 2008, evaluates the awareness of early warning signs and risk factors and barriers to seeking medical advice regarding cancer [22]. Additionally, several modules were developed for specific types of cancer focused on early detection and how it can impact survival [23]. 

Cancer literacy score (CLS) was developed in 2011 by European researchers, and it attempts to define cancer literacy in the European context, as already described [15,24].

Cancer message literacy test (CMLT) is a pair of tools—CMLT Listening (CMLT-L) and CMLT Reading (CMLT-R)—developed in the US and published in 2012. They use messages that simulate real situations of adults’ everyday life and allow the establishment of a link between spoken/oral and written/print literacy and the influence of this link in healthcare decision making and health behavior [25,26]. 

Breast cancer literacy assessment tool (B-CLAT) and cervical cancer literacy assessment tool (C-CLAT), also American (2013), measure the comprehension about breast and cervical cancers [27,28]. 

All these tools cover a wide range of cancer literacy dimensions, but they do not address cancer health literacy as a whole set of aspects, i.e., as a process from knowledge to understanding, and they are primarily focused on perceptions, beliefs and attitudes toward cancer. 

Cancer Health Literacy Test (CHLT-30), developed in the US in 2014, addressed this limitation. CHLT-30 is targeted at cancer patients, focusing on general aspects of cancer, from prevention to treatment, and measuring cancer health literacy along a continuum (not at a specific stage of the disease) [29]. Moreover, the instrument attempts to identify and measure the comprehensive capabilities of individuals belonging to different social groups (in contrast to other tools focused on primary recognition, e.g., underline word recognition or reading proficiency). CHLT-30 has been designed with the aim of assessing individuals with limited health literacy, and this is an innovation. In addition to the original version targeted at adult English speakers (US population), there is a translated and adapted version in Spanish for Latino cancer patients who are not fluent in English (CHLT-30-DKspa, 2016) [30]. There are two more technical advantages of CHLT. It is quick and easy to apply (10–15 min), as well as score (total number of correct answers); it also includes a validated short version (CHLT-6), which can be used by health professionals in a clinical context.

Another important point for this discussion is the need for validation of cultural and linguistic dimensions of the tools. The tools that were described above are validated for the specific contexts where they were developed, and their use under different conditions requires proper adaptation and validation, since health literacy, and specifically cancer literacy, are concepts that cannot be detached from the social realities they belong to (from psychosocial conditions to institutional contexts). The process, and how the participants cope with it, are critical to validate the tool in its context and should prevail above the thorough compliance with generic guidelines. A validated tool, reliable for use in future interventions, must reflect concerns with the psychosocial context of data gathering.

### 1.3. The Portuguese Scenario

The Portuguese national health system is universal and free, and the population is highly homogeneous regarding ethnic and cultural diversity [31]. The available data show that in the case of serious diseases, such as cancer, most patients use the public health system regardless of their socioeconomic condition, while the private health sector is sought by patients with private health insurance, mostly from middle-high classes that can afford the costs, as in other EU countries [32].

Different studies using the Portuguese version of the Health Literacy Survey (HLS-EU PT, 2016) showed inadequate or problematic levels of health literacy in the Portuguese population [33,34]. A detailed analysis of specific themes (healthcare, disease prevention and health promotion) revealed that half or almost half of the participants have low levels of health literacy [33].

These results are in line with a sociocultural enclosure anchored in high illiteracy levels of the Portuguese population for many decades [35,36]. Notwithstanding the recent positive evolution of Portuguese society and culture, several gaps remain when compared with other European countries [37,38,39], especially the distance between the abstract (positive) value of health and the absence of reasonable health literacy skills.

In the cancer care context, health literacy will impact a complex set of interactions between cancer patients, caregivers and all types of health professionals, including physicians, nurses and health-allied professionals, such as radiologists, psychologists, clinical officers and health assistants. The available data and practices showed that high levels of cancer literacy in patients and caregivers, together with enhanced social and communicational skills of health professionals, will contribute to strengthening the interactions between the multiple agents of the cancer care continuum.

Accordingly, our comprehensive approach to investigating the interactions of Portuguese cancer care services is based on in-depth empirical research, with a focus on cancer literacy levels and communication skills, styles and models, aiming to improve the connections and thus positively impact health outcomes [40]. Among the different strategies and tools required (direct observation, formal and informal interviews, surveys and data analysis), we decided to start with the adaptation and validation of an international cancer literacy evaluation instrument to assess Portuguese patients’ cancer literacy levels. A previously validated tool to properly evaluate cancer literacy levels is essential to profile patients and caregivers as part of our global project that aims to understand the communication gap between patients and health professionals in Portuguese cancer care centers [41]. Considering the characteristics of the internationally available tools described above, and since the development of a new measure is a time-consuming process, we selected the English version of the Cancer Health Literacy Test (CHLT-30) developed by Dumenci et al. [29] to evaluate cancer literacy in Portuguese cancer patients and caregivers. This work describes the cultural and linguistic adaptation, as well as the implementation of a pre-test of the instrument in the Portuguese context.

## 2. Materials and Methods

### 2.1. Translation and Adaptation of the CHLT-30 Tool

As discussed above, CHLT-30’s characteristics and goals generally meet our research purposes and the context where the tool is going to be implemented. Nevertheless, adopting the 30 items of the English version of CHLT [29] and applying them in a different sociocultural context required a careful process of language translation and cultural adaptation [42,43]. 

The final version of the tool is available in Supplementary Materials, Questionnaire S1—Cancer Health Literacy Test-30 Portuguese Version (CHLT-30 PT)—and Table S2 (see the Supplementary Materials), with the correspondence for item adaptations between the original and the Portuguese version of CHLT. 

**Figure 1:**
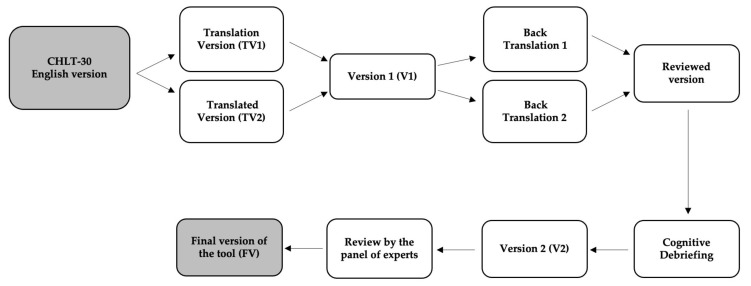
Process of translation and cultural adaptation of the English version of the Cancer Health Literacy Test (CHLT-30) [29] into Portuguese language.

The translation and cross-cultural adaptation of the selected tool were based on the guidelines proposed by Wild et al. [44]. The standardized procedures developed for this process include translation and back-translation, review by a panel of experts of the translation and back-translation, and pre-testing (see Figure 1).

Two independent researchers fluent in Portuguese translated the original version of the instrument, which resulted in translated version 1 (TV1) and translated version 2 (TV2), which were combined in the first version of the translated tool (V1). The process of translation and adaptation included proper names, dishes/foods, measurements (metric system) and some specific issues regarding the institutional procedures (e.g., Portuguese health system mandatory template for prescriptions). 

The next step, back-translation, comprised two independent researchers who back-translated this version to the English language again. From the back-translation, a reviewed version was acquired. Cognitive debriefing was ensured to identify any other problems with the questions. Five adult volunteers of different ages and education levels filled in the questionnaire. They were mainly women with high school or college degrees and aged between 32 and 78 years old and included one researcher and one physician. They all had a cancer diagnosis in the last 5 years. In this step, after the completion of the self-administered version of the questionnaire, participants were encouraged to give feedback about the questionnaire. Additional issues came up and were discussed with the volunteers, such as the organization of questions, the layout of the instrument (including the font size) and questions/concepts that were found difficult to understand. The results from this step were integrated in a second version of the instrument (V2). This version was submitted to a panel of experts composed of a multidisciplinary team of 7 social and health researchers, including oncologists, pathologists and general practitioners. One statistician, one sociologist, as well as other health researchers, were convened to review version (TV2) of the Portuguese version of the instrument. The review conducted by the panel of experts was crucial to ensure the clarity of the final version for the pre-test, final version (FV), with a sample of cancer patients being followed in cancer care units (after the approval of the ethics committee of the hospitals, as required). 

Beyond syntax and semantic issues, the technique underlying this kind of instrument requires careful work on the socio-communication process with the potential respondents [45,46]. As the tool explicitly aims to evaluate literacy and, despite all efforts, it will raise school evaluation issues, it is crucial to pre-guarantee the confidence of the respondents, especially those who will probably reveal difficulties. We included the options “I don’t know” or “I do not want to answer” for all the items (as in CHLT-30-DKspa), thus minimizing the possibility of randomized answers given by the participants. 

### 2.2. Pre-Test of the CHLT-30 PT Tool

The final version (FV) is structured in two parts (see details in Supplementary Materials, Questionnaire S1): (a) 30 items to test cancer literacy, following the original version; (b) 10 items for patients’ sociodemographic characterization. A test–retest was conducted between January and June of 2019. Recruitment took place in the outpatient clinic of two hospitals from the public and the private health system (given the differences mentioned above). Enrolment was voluntary, with the patients having the option to decline participation after filling in the questionnaire. Patients’ eligibility was identified by the nurses according to the following criteria: (a) male/female; (b) age ≥ 18 years old; (c) patients undergoing any kind of treatment; (d) with or without cancer family history. Specific exclusion criteria were applied: (a) pregnancy or breastfeeding women; (b) diagnosed mental illness; (c) palliative treatment/care.

The study was introduced to the patients by the nurses and the field researcher. An information sheet was given to the patients or immediate caregivers. A significant share of the patients decided not to participate (mostly old people), not because of the nature of the object of the study, but because they were not able to self-complete the questionnaire due to reading problems, vision impairments and similar issues. The result was a convenience sample with 71 patients. 

All the participants signed informed consent to participate before the application of the questionnaire. They were clearly instructed about the “don’t know” answer option in each question and advised to choose this option in case of doubt or if they would not know the correct answer.

Questionnaires were applied using a self-administered version (paper or tablet format), and its implementation was performed in the presence of the researcher in the field to provide participants the necessary support when needed. Since we were dealing with cancer patients, all of them undergoing treatment, they were physically and psychologically vulnerable, so the presence of the researcher was of utmost importance for patients to feel comfortable and confident when participating in the study. 

Two implementations were performed for test and retest purposes. An interval of at least two weeks between test and retest was considered to prevent biased answers in the retest due to the eventual recall of questions or previous answers by the participants. 

Data from both tests were analyzed using IBM^®^ SPSS^®^ Statistics, version 25. Descriptive statistics were performed to characterize the sample and obtain the results of this pre-test. Analysis of variance (ANOVA) was also performed in order to identify statistically significant differences between the total score (min.: 0; max.: 30) obtained in the CHLT-30 and gender, age and education level. 

## 3. Results

### 3.1. Patient Characterization

Table 1 details the characterization of the 71 patients enrolled in the test. Almost half were from the public healthcare system (46.5%), mostly females (73.2%), married (56.3%), aged between 23 and 78 years (mean = 50.6) and more than half with a higher education degree (62.0%). Most of the patients were currently undergoing treatment (84.5%), and almost half had been diagnosed less than a year before (45.1%) with different tumor types. In the second application (retest), 30 participants participated (42.3%) in the study, with dropout being due to different reasons, i.e., deterioration of physical condition (e.g., treatment side effects) or reschedule of appointments/treatments. We are dealing with patients in vulnerable conditions, and their clinical condition can change rapidly; therefore, following these patients is a limitation when the study requires different moments of intervention or participation [47].

### 3.2. Internal Reliability

Internal reliability was assessed using Cronbach’s alpha in order to test the degree to which items in a test are interrelated and vary from 0 to 1 [48]. Assessment through Cronbach’s alpha shows a good level of interrelatedness among the items of the test [48]: 0.86 in the test and 0.80 in the retest for the participants that completed the test and retest (N = 30). 

### 3.3. Construct Validity

The patients’ raw score mean (number of correct answers) in both test (23.96) and retest (25.97) and the distribution of scores categories are not statistically different, which shows the validity of the questionnaire (see Table 2). 

The percentage results for all correct answers in the test and retest are presented in Table 3. In the test, the items with a high percentage of correct answers are related to practical aspects of the disease regarding cancer diagnosis (Q9), treatment (Q7, Q25, Q29) and support (Q20). Items with a lower percentage of correct answers are related to applied knowledge about nutrition (Q1), staging of the disease (Q23), risk factors (Q19) and navigation (Q13), which also implies the application of mathematical concepts and numeracy skills. In the retest, similar results were found. The items with higher score were Q9, Q25 and Q29. Items Q1, Q19 and Q23 also revealed lower scores, as well as Q15 and Q24. 

Table 4 presents the dependent sociodemographic characteristics and score categories. We found evidence of a significant association between higher education levels and better scores (data not shown) compared to the original report of CHLT-30-DKspa implementation. No statistically significant associations were found between score categories and gender, age, time since diagnosis or type of health system.

The results from the analysis of variance (ANOVA) regarding the total score in the CHLT-30 PT and sociodemographic characteristics (gender, age and education level categories) revealed that no statistically significant differences were found for gender (*p* = 0.024) and age categories (*p* = 0.442). However, regarding the education level, the analysis indicates statistically significant differences in the total score in at least one of the education level categories (*p* < 0.001). According to the table of multiple comparisons, individuals who had an elementary or middle school level had an average of 5.8 points less in the total score than those with high school level (*p* < 0.001) and 8.3 points less in total score than those with college or higher level (*p* < 0.001). Those who had a high school level had an average of 2.5 points less in total score than those who had college or higher education level (*p* = 0.048). Detailed results of this analysis are available in Supplementary Materials (S1—Summary of the ANOVA analysis). 

Future analysis can also be performed to explore other significant associations in a wider implementation. 

## 4. Discussion

We translated and adapted the original version of CHLT-30 [29] and conducted a pre-test with 71 cancer patients from public and private healthcare systems in order to assess the Portuguese version of CHLT-30.

Our results were consistent and similar to the ones obtained in the validation of the original and translated (CHLT-30DKspa) versions of CHLT-30 [29,30]. CHLT-30 PT presents good internal reliability (0.86), although slightly lower than the other versions (0.88 for both). The observed difference in the retest alpha Cronbach (0.80 vs. 0.90) is possibly due to the smaller size of our sample.

The raw score mean for the 30 tested items with the CHLT-30 PT is similar to the original CHLT-30 (23.57 vs. 23.96). To further detail the analysis of our results, we had adopted the CHLT-30DKspa classification of three score categories (see Table 2). No statistically significant differences were found between test and retest for the score categories, proving the validity of the tool. The obtained results showed that only 2.8% of our patients scored in the low-range category (≤10 correct answers), while 56.4% and 40.8% scored, respectively, in the intermediate (11–26 correct answers) and high-range (≥27 correct answers) categories. These results are statistically different (*p* < 0.001) from the results obtained by Echeverry et al. with CHLT-30DKspa. Briefly, 17.5% of the patients scored in the low-range category, while 73.8% and 8.8% scored, respectively, in the intermediate and high-range categories. This difference might be explained by the patients’ education levels, with our sample having a much lower proportion of participants with less than high school degree (16.9% vs. 39.3%). 

Moreover, the total score is directly related to the individuals’ level of education, which will be a good indicator of construct validity of the CHLT-30 PT, as revealed in the ANOVA analysis. These results are similar to those that were presented in the analysis of CHLT-30 DKspa [30]. This relation is also well reported in the literature. Higher education levels are directly related to better health literacy. 

Overall, we can affirm that these preliminary results are consistent with the ones obtained in the validation of the other available versions of the CHLT-30, the English and the Spanish versions. The Portuguese version of the instrument is now ready for the next steps of the validation process.

The implementation of cancer literacy questionnaires must consider multiple factors affecting the articulation between the object and the instrument used to address it [47]. We opted for a self-administered paper or tablet format, both in written mode, due to time and space constraints in the cancer care units of the two hospitals. CHLT-30 and CHLT-30DKspa reading mode options were available through touchscreen devices (CHLT-30) and a combination of self-administered and interviewer-administrated modes (CHLT-30DKspa). In order to include patients with visual impairments and reading/writing difficulties, avoiding hindering self-completion of the questionnaire and participation constraints, future implementations of the CHLT-30 PT should be adapted for an interviewer-administrated format (as occurred with the Spanish version of the original tool [30]). 

### Strengths and Limitations

The results obtained in the pre-test of the Portuguese version of the tool clearly reinforce the importance and need for a tool to measure cancer literacy in the Portuguese population.

Working closely with the patients is a major strength of this work, favoring their involvement and willingness to share the necessary information on cancer literacy. Personal contact with the patients is crucial to engage them in the research, especially when we are dealing with cancer patients who are in a vulnerable physical and psychological condition due to the burden of the disease and treatments. It was an important step in ensuring the patients’ recognition of the value and importance of these studies and how they are expected to positively impact the future of cancer care, which in turn means making the participants aware of their essential role; the research could never be accomplished without their contribution.

Therefore, the fieldwork is challenging due to the limitation of patients’ availability. Cancer patients deal with a huge physical and emotional burden, and despite the willingness to participate in the study, the daily routine of the treatments and their side effects, as well as the high number of medical appointments and procedures intrinsic to the progress of the disease, objectively condition their availability to participate. Data collection being dependent on the patients’ physical and emotional condition on a daily basis impacts the sample size, so a trade-off between (higher) size and (lower) data quality must take place.

The current scenario of the COVID-19 pandemic is drastically limiting the research that we are conducting with cancer patients. The necessary fieldwork with cancer patients in order to collect crucial information on cancer literacy is severely restricted, as social distancing is required and social interactions with risk groups (cancer patients) should be minimized. 

We are dealing with unique circumstances. On the one hand, health literacy is critical to fighting COVID-19, as behavioral measures are proved to be effective in containing the virus from spreading [49]. On the other hand, patient literacy education is almost entirely supported through in-person interaction with health professionals [50]. So, in times of pandemics, we must implement new solutions to work “closely” with cancer patients while respecting social distancing. This is of the utmost importance regarding vulnerable social groups, especially the elder ones, who have a high probability of disease disability and are less familiar with the use of information and communication technologies. 

## 5. Conclusions

In conclusion, the available cancer health literacy assessment tool CHLT-30 was successfully translated and adapted into a Portuguese version for cancer patients. 

Fieldwork suggests the future adaptation of this tool into an interview script to facilitate data collection while accommodating participants’ needs and limitations. This work took place before the COVID-19 pandemic context. We need to find new strategies to cope with present restrictions regarding face-to-face interactions, which will strongly condition the fieldwork. This is a critical issue, as future steps of our research should include an extended field test (pilot test) to fully validate the Portuguese version of CHLT-30 PT. 

The validation of the short version of the tool (CHLT-6) will also be included in the future steps in order to promote its use by healthcare professionals in their clinical practice. 

The adaptation of this tool for the Portuguese population and its implementation in clinical practice will allow the characterization of patients’ cancer literacy along the whole cancer care continuum, facilitating the identification and implementation of adequate socio-educational strategies. This study is part of a more comprehensive project that focuses not only on the characterization of patient’s cancer literacy levels but also on health professionals’ communication skills and styles, aiming to simultaneously provide educational content and efficient communication models, which will enhance interactions in cancer care services and consequently improve health outcomes.

Future directions for cancer literacy research must include new paths to deliver innovative patient literacy programs. Blended models, merging the strengths of in-person and digital learning, will lead to expanding literacy empowerment, reducing disparities and ultimately improving health outcomes [50].

## Figures and Tables

**Table 1:** Sociodemographic and clinical profile of patients’ sample (*n* = 71).

Characteristics		Result (%)
Age	20–35 years	13 (18.3)
	36–50 years	20 (28.2)
	51–65 years	24 (33.8)
	>65 years	12 (16.9)
	No answer	2 (2.8)
Gender	Male	19 (26.8)
	Female	52 (73.2)
Marital Status	Married/Partnership	44 (62.0)
	Divorced/Separated	10 (14.1)
	Single	12 (16.9)
	Widowed	4 (5.6)
	No answer	1 (1.4)
Education	Elementary/Middle school	12 (16.9)
(Highest qualification obtained)	High school	15 (21.1)
	College or higher degree	44 (62.0)
Healthcare System	Public	33 (46.5)
	Private	18 (25.4)
	No answer	20 (28.2)
Cancer Type	Breast	12 (16.9)
	Colon/Rectal/Anal	10 (14.1)
	Endocrine	3 (4.2)
	Genitourinary	3 (4.2)
	Hematologic	4 (5.6)
	Kidney	1 (1.4)
	Lung	2 (2.8)
	Lymphoma	4 (5.6)
	Other	1 (1.4)
	Stomach	3 (4.2)
	Unknown/Not Defined	28 (39.4)
Time since the diagnosis	<1 year	32 (45.1)
	1–2 years	12 (16.9)
	3–5 years	15 (21.1)
	>5 years	10 (14.1)
	No answer	2 (2.8)

**Table 2:** Score categories: Test vs. Retest.

Score Categories ^1^	Test (N = 71)		Retest (N = 30)	
	N	%	N	%
Low range (≤10 correct answers)	2	2.8	---	---
Intermediate range (11–26 correct answers)	40	56.4	13	43.3
High range (≥27 correct answers)	29	40.8	17	56.7

^1^ Previously described in CHLT-30-DKspa [30].

**Table 3:** Questionnaire items—correct answers.

	Correct Answers			
	Test (N = 71)		Retest (N = 30)	
Item	N	%	N	%
Q1. High calorie	27	38.0	14	46.7
Q2. Next pill	61	85.9	29	96.7
Q3. Chemotherapy	63	88.7	28	93.3
Q4. Hemoglobin range	63	88.7	29	96.7
Q5. Oral cancer	54	76.1	24	80.0
Q6. Side effects	55	77.5	25	83.3
Q7. Risk of complications	67	94.4	29	96.7
Q8. Palliative care	60	84.5	25	85.3
Q9. Biopsy	68	95.8	30	100.0
Q10. Appointment location	53	74.6	26	86.7
Q11. Body temperature	58	81.7	29	96.7
Q12. Stage 1 cancer	58	81.7	28	93.3
Q13. Direction	47	66.2	25	83.3
Q14. Efficacy	64	90.1	27	90.0
Q15. Tumor spread	49	69.0	22	73.3
Q16. Generic drugs	56	78.9	25	83.3
Q17. Survival rate	52	73.2	25	83.3
Q18. Fasting	59	83.1	25	83.3
Q19. Smoking risk	46	64.8	23	76.7
Q20. Physical therapist	67	94.4	26	86.7
Q21. Inoperable tumor	63	88.7	29	96.7
Q22. High-fiber food	61	85.9	29	96.7
Q23. Metastasized	43	60.6	18	60.0
Q24. Benign tumor	50	70.4	21	70.0
Q25. Radiation treatment	69	97.2	30	100.0
Q26. Complication rate	54	76.1	27	90.0
Q27. Double dose	58	81.7	26	86.7
Q28. Book chapter	55	77.5	28	93.3
Q29. Dose time	65	91.5	30	100.0
Q30. Map reading	56	78.9	27	90.0

**Table 4:** Sociodemographic characteristics and score categories.

			Score Categories			
	Low Range		Intermediate Range		High Range	
Characteristic	N	%	N	%	N	%
Health System						
Public	1	50.00	20	50.00	12	41.38
Private	1	50.00	9	22.50	8	27.59
No answer	0	0	11	27.50	9	31.03
Time since diagnosis						
<1 year	1	50.00	21	52.50	10	34.48
1–2 years	0	0.00	7	17.50	5	17.24
3–5 years	1	50.00	7	17.50	7	24.14
>5 years	0	0.00	4	10.00	6	20.69
No answer	0	0.00	1	2.50	1	3.45
Education						
Elementary or Middle School	1	50.00	10	25.00	1	3.45
High School	0	0.00	12	30.00	3	10.35
College or higher degree	1	50.00	18	45.00	25	86.20
Age						
20–35	0	0.00	9	23.08	4	13.79
36–50	0	0.00	10	25.64	10	34.48
51–65	1	100.0	14	35.90	9	31.03
>65	0	0.00	6	15.38	6	20.70
Gender						
Male	2	100.00	10	25.00	7	24.14
Female	0	0.00	30	75.00	22	75.86

## Data Availability

The dataset supporting the conclusions of this article is available from the corresponding author on reasonable request.

## Supplementary Materials

The following materials are available online at https://www.mdpi.com/article/10.3390/ijerph19106237/s1, Table S1: Summary of instruments to measure cancer literacy, Table S2: Items adaptations—correspondence between the original and new versions of CHLT, Questionnaire S1: Cancer Health Literacy Test—30 Portuguese Version, CHLT-30 PT, S1: Summary of the ANOVA analysis.
