# Supplementary material for: Translation and Cross-Cultural Adaptation of the Cancer Health Literacy Test for Portuguese Cancer Patients: A Pre-Test
Source: Int J Environ Res Public Health. 2022 May 20;19(10):6237. doi: 10.3390/ijerph19106237 (PMC9141979; doi:10.3390/ijerph19106237)
Supplement: Supplementary file 1 [file ijerph-19-06237-s001.zip › S1.pdf]

Supplementary material S1: Summary of ANOVA analysis (Gender, Age and Education)

Gender

Descriptives

Total Score

	N	Mean	Std. Deviation	Std. Error	95% Confidence Interval for Mean		Minimum	Maximum
					Lower Bound	Upper Bound		
Male	19	22.74	6.935	1.591	19.39	26.08	5	30
Female	52	24.40	4.221	0.585	23.23	25.58	11	29
Total	71	23.96	5.089	0.604	22.75	25.16	5	30

ANOVA

Total Score

	Sum of Squares	df	Mean Square	F	Sig.
Between Groups	38,670	1	38,670	1.504	0.224
Within Groups	1,774,203	69	25,713		
Total	1,812,873	70			

Age

Descriptives

Total Score

	N	Mean	Std. Deviation	Std. Error	95% Confidence Interval for Mean		Minimum	Maximum
					Lower Bound	Upper Bound		
20–35	13	22.92	4.645	1.288	20.12	25.73	15	28
36–50	20	25.65	2.943	0.658	24.27	27.03	16	29
51–65	24	23.83	5.843	1.193	21.37	26.30	5	29
>65	12	23.92	5.468	1.579	20.44	27.39	14	30
Total	69	24.20	4.859	0.585	23.04	25.37	5	30

ANOVA

Total Score

	Sum of Squares	df	Mean Square	F	Sig.
Between Groups	67,436	3	22,479	0.950	0.422
Within Groups	1,537,723	65	23,657		
Total	1,605,159	68			

Multiple Comparisons

Dependent Variable: Total Score

Bonferroni

(I) Age	(J) Age	Mean Difference (I-J)	Std. Error	Sig.	95% Confidence Interval	
					Lower Bound	Upper Bound
20–35	36–50	−2.727	1.733	0.722	−7.44	1.99
	51–65	−0.910	1.675	1.000	−5.47	3.65
	>65	−0.994	1.947	1.000	−6.29	4.31
36–50	20–35	2.727	1.733	0.722	−1.99	7.44
	51–65	1.817	1.473	1.000	−2.19	5.82
	>65	1.733	1.776	1.000	−3.10	6.57
51–65	20–35	0.910	1.675	1.000	−3.65	5.47
	36–50	−1.817	1.473	1.000	−5.82	2.19
	>65	−0.083	1.720	1.000	−4.76	4.60

>65	20–35	0.994	1.947	1.000	−4.31	6.29
	36–50	−1.733	1.776	1.000	−6.57	3.10
	51–65	0.083	1.720	1.000	−4.60	4.76

Education

Descriptives

Total Score

					95% Confidence Interval for Mean			
	N	Mean	Std. Deviation	Std. Error	Lower Bound	Upper Bound	Minimum	Maximum
Elementary/Middle school	12	17.58	4.776	1.379	14.55	20.62	10	26
High school	15	23.40	3.942	1.018	21.22	25.58	15	29
College or higher degree	44	25.89	4.001	0.603	24.67	27.10	5	30
Total	71	23.96	5.089	0.604	22.75	25.16	5	30

ANOVA

Total Score

	Sum of Squares	df	Mean Square	F	Sig.
Between Groups	655,925	2	327,962	19.276	<0.001
Within Groups	1,156,948	68	17,014		
Total	1,812,873	70			

Multiple Comparisons

Dependent Variable: Total Score

						95% Confidence Interval	
	(I) Education	(J) Education	Mean Difference (I-J)	Std. Error	Sig.	Lower Bound	Upper Bound
LSD	Elementary/Middle school	High School	−5.817 *	1.598	<0.001	−9.00	−2.63
		College or higher	−8.303 *	1.343	<0.001	−10.98	−5.62
	High school	Elementary/Middle school	5.817 *	1.598	<0.001	2.63	9.00
		College or higher	−2.486 *	1.233	0.048	−4.95	−0.03
	College or higher degree	Elementary/Middle school	8.303 *	1.343	<0.001	5.62	10.98
		High school	2.486 *	1.233	0.048	0.03	4.95

*. The mean difference is significant at the 0.05 level.
