# Supplementary material for: Translation and Cross-Cultural Adaptation of the Cancer Health Literacy Test for Portuguese Cancer Patients: A Pre-Test
Source: Int J Environ Res Public Health. 2022 May 20;19(10):6237. doi: 10.3390/ijerph19106237 (PMC9141979; doi:10.3390/ijerph19106237)
Supplement: Supplementary file 1 [file ijerph-19-06237-s001.zip › Supplementary material Questionnaire S1.pdf]

Cancer Health Literacy Test – 30 Portuguese Version, CHLT-30 PT

1. Os médicos recomendam frequentemente, para doentes oncológicos em tratamento, a ingestão de alimentos ricos em proteínas e em calorias. Dos seguintes alimentos qual é o que tem mais proteínas e calorias?

   a. Batatas fritas
   b. Hambúrguer com queijo
   c. Ovo cozido
   d. Não sei/Não quero responder

2.

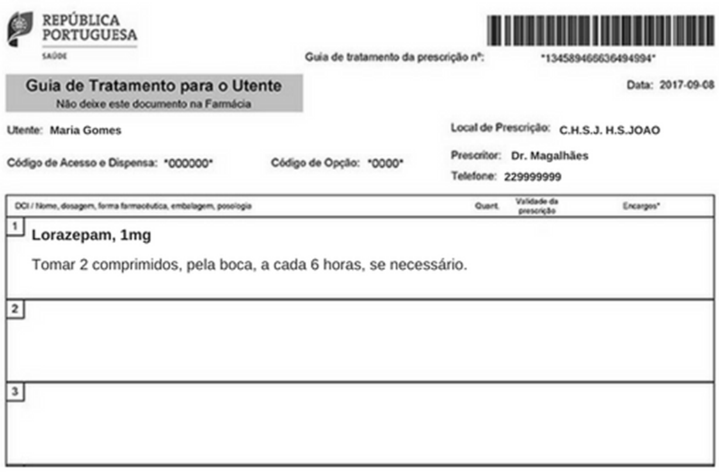

REPÚBLICA PORTUGUESA
SAÚDE

Guia de tratamento da prescrição nº: *134589466636494994*

Guia de Tratamento para o Utente
Não deixe este documento na Farmácia

Data: 2017-09-08

Utente: Maria Gomes

Local de Prescrição: C.H.S.J. H.S.JOAO

Código de Acesso e Dispensa: *000000*

Código de Opção: *0000*

Prescritor: Dr. Magalhães

Telefone: 229999999

DCI / Nome, dosagem, forma farmacêutica, embalagem, posologia	Quant.	Validade da prescrição	Encargos*
1 **Lorazepam, 1mg**<br>Tomar 2 comprimidos, pela boca, a cada 6 horas, se necessário.			
2			
3			

A Maria tomou 2 comprimidos de Lorazepam 1mg às 14h00. A que horas poderá fazer a próxima toma destes comprimidos?

   a. 18h00
   b. 19h00
   c. 20h00
   d. Não sei/Não quero responder

3. A terapia adjuvante é o tratamento do cancro que se dá frequentemente depois da remoção cirúrgica de um tumor. A terapia neoadjuvante é o tratamento que se dá, frequentemente, antes da cirurgia para diminuir o tamanho do tumor.
   O Sr. Silva foi operado para remover o seu tumor. Após a cirurgia, vai fazer quimioterapia. Neste caso, a quimioterapia é:
   a. Neoadjuvante
   b. Adjuvante
   c. Não sei/Não quero responder

4. Os valores normais para a hemoglobina num homem variam entre **13,0 – 17,5** g/dL. A hemoglobina do Sr. Silva é de 9,7g/dL. A hemoglobina do Sr. Silva está dentro do intervalo normal?
   a. Sim
   b. Não
   c. Não sei/Não quero responder

5. Nas pessoas que desenvolvem cancro oral, 25% desses casos ocorrem na língua. O cancro oral ocorre na língua em:
   a. 1 em cada 25 casos
   b. 25 em cada 100 casos
   c. 25 em cada 1.000 casos
   d. Não sei/Não quero responder

6. Efeitos secundários possíveis do Tamoxifeno:

Mais de 30% dos doentes manifestam:
- Calores
- Edemas (ex: tornozelos inchados)
- Corrimento vaginal
- Perda do desejo sexual

Entre 10 a 30% dos doentes manifestam:
- Náuseas
- Irregularidades menstruais
- Alterações do humor
- Perda de peso

Dos seguintes efeitos secundários, qual o mais comum nos doentes que tomam o Tamoxifeno?

a. Edemas
b. Perda de peso
c. Não sei/Não quero responder

7. O tratamento de quimioterapia A tem uma taxa de sucesso de 92% e uma taxa de complicações, a longo prazo, de 15,5%. O tratamento B tem uma taxa de sucesso de 95,9% e uma taxa de complicações, a longo prazo, de 3,8%.
Qual o tratamento que tem menor risco de complicações a longo prazo?

a. Tratamento A
b. Tratamento B
c. Não sei/Não quero responder

8. O objetivo dos cuidados paliativos é curar o cancro.

a. Verdadeiro
b. Falso
c. Não sei/Não quero responder

9. Uma biópsia a um tumor é realizada para:

a. Remover o tumor
b. Diagnosticar o tumor
c. Tratar o tumor
d. Não sei/Não quero responder

Marcação de Consulta Externa

ROTINA

Nº Processo: **12345678**

12345678

No edifício Pav.Cons.Externas; Local/sala K4 - Sala 204.

Exmo(a). Sr(a). MARIA ALMEIDA

Informa-se V. Exª de que tem consulta marcada para quinta-feira. 19 de julho de 2018 às 15:40, para o(a) Dr(a) de C. ONCOLOGIA MÉDICA
DEVERÁ COMPARECER NA MORADA ACIMA INDICADA e apresentar-se 20 minutos antes da hora marcada.
No caso de consulta de Oftalmologia o acesso será através do Atrium Hospitalidade (frente do Hospital S. João – piso 1).
Caso tenha consulta no setor K1 a K6, o acesso deverá ser feito pela Rua Dr. Plácido da Costa, entrada traseira do Hospital.
Caso tenha consulta em Medicina Física e Reabilitação, deverá dirigir-se ao Piso 01 (no interior do Hospital).
Caso tenha consulta de Otorrino ou no setor K7, o acesso deverá ser feito pela Rua Dr. Roberto Frias, portal lateral do Hospital/INEM.

Deverá levar este documento comprovativo de marcação.

Nota: a) Pede-se o favor de trazer os exames efetuados e os medicamentos em uso.
b) Se não puder comparecer, por favor informe o Hospital antecipadamente , entre as 09 e as 17 horas.
c) Lembramos V. Exa. que o pagamento das taxas moderadoras deve ser efetuado no dia da realização da consulta.

PORTO, sexta-feira, 22 de junho de 2018

O MÉDICO

(António Ferreira)

10. Acima está representada a convocatória para a consulta de oncologia médica da Maria. Onde se deve dirigir a Maria para ir para a consulta?
- **a.** Ao Atrium Hospitalidade (frente do Hospital S. João – piso 1)
- **b.** À Rua Dr. Plácido da Costa (entrada traseira do Hospital)
- **c.** À Rua Dr. Roberto Frias (portal lateral do Hospital/INEM)
- **d.** Não sei/Não quero responder

11. A febre medida na axila pode ser classificada da seguinte forma:

Sem febre: entre 36°C e 37,5°
Subfebril: entre 37,5°C e 37,9°C
Febre: entre 38,0°C e 41,1°C
Hiperpirexia: superior a 41,1°C

(Alerta: Se a sua temperatura corporal for igual ou superior a 38°C deve contactar de imediato a linha SNS24 ou o seu médico assistente)

O Sr. Silva tem uma temperatura corporal de 37,9°C. De acordo com a informação fornecida ele deve contactar o seu médico?
- **a.** Sim
- **b.** Não
- **c.** Não sei/Não quero responder

12. Se um doente tem um cancro classificado em estadio I, significa que:
- **a.** O tumor está localizado apenas numa zona limitada
- **b.** O tumor atingiu os órgãos vizinhos
- **c.** O tumor espalhou-se para outros órgãos distantes
- **d.** Não sei/Não quero responder

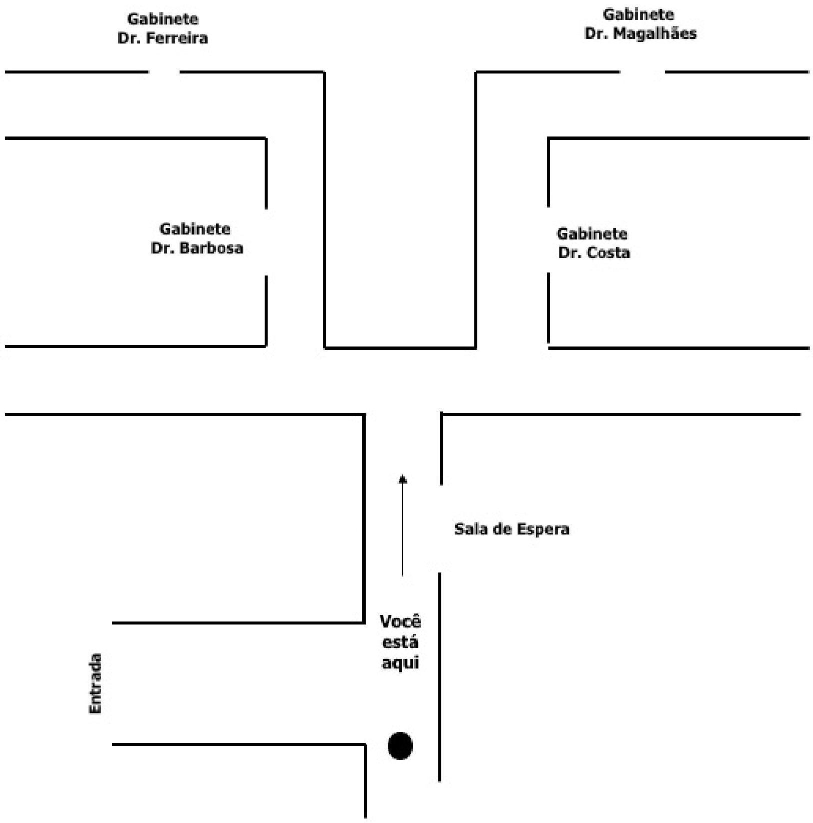

13. A Maria precisa de indicações para chegar ao local da primeira consulta. A rececionista pede que ela vá até ao fundo do corredor e vire à direita, depois vire à esquerda e procure a primeira porta à esquerda.
    Se seguir estas instruções onde irá ter a Maria.
    - **a.** Gabinete Dr. Ferreira
    - **b.** Gabinete Dr. Magalhães
    - **c.** Gabinete Dr. Costa
    - **d.** Não sei/Não quero responder

14. O maior ou menor efeito benéfico de um medicamento traduz a sua:
    - **a.** Impotência
    - **b.** Eficácia
    - **c.** Destreza
    - **d.** Não sei/Não quero responder

15. Durante a cirurgia, expor um tumor ao ar faz com que o tumor se espalhe.
    - **a.** Verdadeiro
    - **b.** Falso
    - **c.** Não sei/Não quero responder

16. Os medicamentos de marca têm os mesmos princípios ativos que os medicamentos genéricos, mas com um pequeno extra, que os torna melhores.
    - **a.** Verdadeiro
    - **b.** Falso
    - **c.** Não sei/Não quero responder

17. A taxa de sobrevivência global a cinco anos em doentes com cancro da próstata é de 98%. Isso significa que 5 anos após o tratamento, 98% dos doentes com cancro da próstata estarão:
    - **a.** Vivos
    - **b.** Livres da doença
    - **c.** Falecidos

   d. Não sei/Não quero responder

18. A convocatória diz para não comer ou beber 9 horas antes da consulta. A Maria tem uma consulta às 11h15m de sexta-feira. A partir de que horas não deverá ela comer nem beber.
   a. quinta-feira às 23h15m
   b. sexta-feira à 1h15m
   c. sexta-feira às 2h15m
   d. Não sei/Não quero responder

19. Os cientistas estimam que o tabaco é responsável por 85% a 90% das mortes por cancro do pulmão. Isto significa que 85% a 90% dos fumadores terá cancro do pulmão.
   a. Verdadeiro
   b. Falso
   c. Não sei/Não quero responder

20. O papel do fisioterapeuta é conversar com o doente sobre as suas necessidades emocionais.
   a. Verdadeiro
   b. Falso
   c. Não sei/Não quero responder

21. Um tumor é considerado "inoperável" quando não pode ser tratado com:
   a. Radioterapia
   b. Cirurgia
   c. Quimioterapia
   d. Não sei/Não quero responder

22. Os doentes que estão a fazer radioterapia devem comer alimentos ricos em fibras e evitar alimentos com muitos condimentos, cafeína ou lacticínios. Qual dos seguintes alimentos é melhor para comer quando se faz radioterapia?
   a. Frango com piripíri
   b. Gelado
   c. Banana
   d. Não sei/Não quero responder

23. Quando o cancro está metastizado significa que:
   a. Se espalhou para outras partes do corpo
   b. Se espalhou para outras partes do órgão onde surgiu
   c. Parou de se "espalhar"
   d. Não sei/Não quero responder

24. Um tumor benigno é cancro.
   a. Verdadeiro
   b. Falso
   c. Não sei/Não quero responder

25. A Maria fará radioterapia uma vez por dia, de segunda a sexta-feira. Se a Maria efetuar o tratamento durante 4 semanas, quantas vezes irá receber a radioterapia?
   a. 5
   b. 10
   c. 20
   d. Não sei/Não quero responder

26. Das 100 pessoas que recebem tratamento, espera-se que metade responda ao tratamento. Dos que respondem, espera-se que metade tenha complicações. Das pessoas que respondem ao tratamento quantas se espera que tenham complicações?
   a. 25
   b. 35
   c. 50
   d. Não sei/Não quero responder

27. Se os doentes melhoram ao tomar o medicamento B duas vezes por dia, se tomarem o medicamento B 3 vezes ao dia melhorarão mais rapidamente.

a. Verdadeiro
b. Falso
c. Não sei/Não quero responder

28.

Índice		
Capítulo 1	O que é o cancro?	p. 4
Capítulo 2	Opções de tratamento	p. 52
Capítulo 3	Falar com os outros sobre o cancro	p. 86

Se a Maria quiser aprender mais sobre os efeitos secundários da radiação, qual é o capítulo onde será mais provável encontrar informação sobre o tema?

a. Capítulo 1
b. Capítulo 2
c. Capítulo 3
d. Não sei/Não quero responder

29. A Maria toma o seu primeiro comprimido às 10h00. Se ela tem que tomar este medicamento de 4 em 4 horas, a que horas terá de fazer a 3ª toma deste medicamento?

a. 14h00m
b. 16h00m
c. 18h00m
d. Não sei/Não quero responder

30.

▲	Radiação	Endoscopia	▲
◀	Quartos 200-300	Quartos 100-200	▶
◀	Quartos 400-500	Quartos 300-400	▶

Se o Sr. Silva precisa de ir ter com o seu médico ao quarto 202, qual a direção que deve seguir?

a. Em frente
b. à direita
c. à esquerda
d. Não sei/Não quero responder
