# Supplementary material for: Translation and Cross-Cultural Adaptation of the Cancer Health Literacy Test for Portuguese Cancer Patients: A Pre-Test
Source: Int J Environ Res Public Health. 2022 May 20;19(10):6237. doi: 10.3390/ijerph19106237 (PMC9141979; doi:10.3390/ijerph19106237)
Supplement: Supplementary file 1 [file ijerph-19-06237-s001.zip › Supplementary material Table S1.pdf]

Table S1. Summary of instruments to measure cancer literacy.

Instrument	Author(s)	Year of publication	Description	Domains/Themes	Validation sample population age:	Number of items:	Sample size in validation study:	Modes of administration	Approximate administration time (minutes)
Cancer Awareness Measure – CAM	Stubbings et al.	2009	Standardized measurement tool to assess cancer awareness, explore risk factors for poor cancer awareness, and develop and evaluate interventions to promote cancer awareness.	- Warning signs<br>- Seeking help<br>- Barriers to seeking help<br>- Risk factors<br>- Cancer and age<br>- Most common cancers<br>- NHS screening programmes	Adults: >=18 yrs	47	148	Face to face or telephone interview<br>Online, paper self-complete or mailed survey	n/a*
The Cancer Literacy Score – CLS	Diviani, N. & Schulz, P.J.	2012	A comprehensive measure of a cancer literacy of the concept to go in the direction of a context- and content-specific concept of health literacy.	Prose: Comprehension<br>An index formed by 37 knowledge items regarding different aspects of cancer:<br>- cancer risk<br>- detection and diagnosis<br>- treatment<br>- coping and information	Adolescents and adults: >=15 yrs	37	639	Face-to-face interview;<br>Paper and pencil	n/a*
Cancer Message Literacy Test-Reading – CMLT-Reading	Mazor et al.	2012	The CMLT-Reading assesses comprehension of written messages on cancer prevention and screening.	Prose: Comprehension, Information seeking: Document	Adults: 40 to 70 years	23	1074	Self-administered;<br>Paper and pencil	10
Cancer Message Literacy Test-Listening – CMLT-Listening	Mazor et al.	2012	Assesses comprehension of spoken messages related to cancer prevention and screening,	Prose: Comprehension;<br>Communication: Listener	Adults: 40 to 70 years	48	1074	Self-administered;<br>computer-based	60
Breast Cancer Literacy Assessment Tool - B-CLAT	Williams, Reckase, & Rivera-Vasquez;	2008	Breast cancer literacy assessment that measure functional literacy	Prose: Comprehension;<br>It combined a multiple-choice and true/false format, and it had three domains:<br>- cancer awareness<br>- knowledge of screening<br>- modalities prevention and control	Adults: 27 to 64 years	16	16	Verbal administration by lay people	10
	Williams, K.P., Templin, T.N., & Hines, R.D.	2013			Adults: 20 to 102 years	21	543	Verbal administration by lay people	---
Cervical Cancer Literacy Assessment Tool - C-CLAT	Williams, Reckase, & Rivera-Vasquez	2008	Cervical cancer literacy assessment that measure functional literacy	Prose: Comprehension;<br>Three content domains:<br>- awareness<br>- knowledge<br>- prevention/control	Adults: 27 to 64 years	12	16	Verbal administration by lay people	10
	Williams, K.P., & Templin, T.N	2013			Adults: 20 to 102 years	16	543	Verbal administration by lay people	---
30-Item Cancer Health Literacy Test – CHLT-30	Dumenci et al.	2014	Instrument designed to measure cancer health literacy along a continuum	Prose: Comprehension, Numeracy	Adults: 18 to 93 years	30	1306	Computer-based;<br>Administration using	10 - 15

								touchscreen devices	

Table S1. Cont.

Instrument	Measure validation		Reliability	Translation		Other Versions
	**Language of validated version:**	**Country where validated:**	**Test-retest correlation Cronbach's alpha:**	**Translated/Adapted versions**	**Language**	
Cancer Awareness Measure (CAM)	English	United Kingdom	Cronbach's alpha = 0.77	Breast Cancer Awareness Measurement Tool in Malaysia (B-CAM-M) (Htay et al. 2020)	Malay	1. CAM – modified version used since 2014<br>2. Bowel Cancer CAM<br>3. Breast Cancer CAM<br>4. Cervical Cancer CAM<br>5. Lung Cancer CAM<br>6. Ovarian Cancer CAM
				Arabic Breast Cancer Awareness Measure (Al-Khasawneh et al 2016)	Arabic	
				Persian version of the Breast Cancer Awareness Measure (BCAM) (Heidari & Feizi 2018)	Persian	
The Cancer Literacy Score - CLS	Italian	Switzerland	Cronbach's alpha = 0.769<br>Four-week test-retest reliability (r = .721, p < .001)	---	---	---
Cancer Message Literacy Test-Reading - CMLT-Reading	English	United States of America	Cronbach's alpha = 0.75	---	---	---
Cancer Message Literacy Test-Listening - CMLT-Listening	English	United States of America	Cronbach's alpha = 0.84	---	---	---
Breast Cancer Literacy Assessment Tool - B-CLAT	English	United States of America	Cronbach's alpha = 0.85	Spanish and Arabic version of the Breast Cancer Literacy Assessment Tool - B-CLAT (Rivera-Vasquez et al. 2009)	Spanish<br>Arabic	---
	English<br>Spanish<br>Arabic		Cronbach's alpha = 0.73	---	---	---
Cervical Cancer Literacy Assessment Tool - C-CLAT	English	United States of America	Cronbach's alpha = 0.87	Spanish and Arabic version of the Cervical Cancer Literacy Assessment Tool - C-CLAT (Rivera-Vasquez et al. 2009)	Spanish<br>Arabic	---
	English<br>Spanish<br>Arabic		Cronbach's alpha = 0.72	---	---	---

30-Item Cancer Health Literacy Test - CHLT-30	English	United States of America	Cronbach's alpha = 0.88<br>2 -week retest = 0.90<br>6 mo. Retest = 0.92	Cancer Health Literacy Test – 30 – Spanish – CHLT-30-DKSpa (Echeverri, Anderson & Nápoles, 2016)	Spanish	6-Item Cancer Health Literacy Test - CHLT-6 (Instrument designed to determine whether a patient has limited cancer health literacy)

*n/a – Information not available
