# Supplementary material for: Translation and Cross-Cultural Adaptation of the Cancer Health Literacy Test for Portuguese Cancer Patients: A Pre-Test
Source: Int J Environ Res Public Health. 2022 May 20;19(10):6237. doi: 10.3390/ijerph19106237 (PMC9141979; doi:10.3390/ijerph19106237)
Supplement: Supplementary file 1 [file ijerph-19-06237-s001.zip › Supplementary material Table S2.pdf]

Table S2. Items adaptations - correspondences between the original and new versions of the CHLT.

	Items adaptations	
	CHLT-30<br>(original version)	CHLT-30 PT<br>(Portuguese version)
**Q1. High calorie**	a) *"French fries"*	Replaced by the name of popular dishes or food with similar characteristics to the options presented in the original version:<br>a) *"Batata frita"*
**Q2. Next pill**	Figure that illustrates medicine prescription.<br>12-hour time format (AM-PM):<br>*a) 6 p.m.*<br>*b) 7 p.m.*<br>*c) 8 p.m.*	Replaced using an example with the mandatory template of Portuguese medical prescriptions.<br>Converted to 24-hour time format:<br>*a) 18h00*<br>*b) 19h00*<br>*c) 20h00*
**Q3. Chemotherapy**	---	---
**Q4. Hemoglobin range**	*"The normal range for hemoglobin for a male is 13.3 - 17.2 g/dl."*	The normal range was modified according to the Portuguese Directorate-General of Health guidelines.<br>*"The normal range for hemoglobin for a male is 13.0 - 17.5 g/dl."*
**Q5. Oral cancer**	---	---
**Q6. Side effects**	---	---
**Q7. Risk of complications**	---	---
**Q8. Palliative care**	---	---
**Q9. Biopsy**	---	---
**Q10. Appointment location**	Figure that illustrates an appointment card.	Replaced using an example according to the hospital facilities and services organization where the test has been conducted.
**Q11. Body temperature**	Fahrenheit degrees (ºF)	Converted to Celsius degrees (ºC)
**Q12. Stage 1 cancer**	---	---
**Q13. Direction**	---	---
**Q14. Efficacy**	---	---
**Q15. Tumor spread**	---	---
**Q16. Generic drugs**	---	---
**Q17. Survival rate**	---	---
**Q18. Fasting**	12-hour time format (AM-PM):<br>*a) Thursday at 11:15 p.m.*<br>*b) Friday at 1:15 a.m.*<br>*c) Friday at 2:15 a.m.*	Converted to 24-hour time format:<br>*a) quinta-feira às 23h15m*<br>*b) sexta-feira à 1h15m*<br>*c) sexta-feira às 2h15m*
**Q19. Smoking risk**	---	---
**Q20. Physical therapist**	---	---
**Q21. Inoperable tumor**	---	---
**Q22. High fiber food**	a) Curry	Replaced by the name of popular dishes or food with similar characteristics to the options presented in the original version:<br>a) Frango com piripíri ("spicy

		chicken")
**Q23. Metastasized**	---	---
**Q24. Benign tumor**	---	---
**Q25. Radiation treatment**	---	---
**Q26. Complication rate**	---	---
**Q27. Double dose**	---	---
**Q28. Book chapter**	---	---
**Q29. Dose time**	12-hour time format (AM-PM):<br>*a) 2:00 p.m.*<br>*b) 4:00 p.m.*<br>*c) 6:00 p.m.*	Converted to 24-hour time format:<br>*a) 14h00m*<br>*b) 16h00m*<br>*c) 18h00m*
**Q30. Map reading**	---	---

Note: We used the same item numbers and condensed labels from the original study to refer to each item. This table illustrates the items that were not only translated but also the items that imply a cultural and social adaptation to the Portuguese context.
The "I don't know"/"I do not want to answer" option was added to all the questions.
